# The first genetic landscape of inherited retinal dystrophies in Portuguese patients identifies recurrent homozygous mutations as a frequent cause of pathogenesis

**DOI:** 10.1093/pnasnexus/pgad043

**Published:** 2023-02-13

**Authors:** Virginie G Peter, Karolina Kaminska, Cristina Santos, Mathieu Quinodoz, Francesca Cancellieri, Katarina Cisarova, Rosanna Pescini Gobert, Raquel Rodrigues, Sónia Custódio, Liliana P Paris, Ana Berta Sousa, Luisa Coutinho Santos, Carlo Rivolta

**Affiliations:** Institute of Molecular and Clinical Ophthalmology Basel (IOB), Basel 4031, Switzerland; Department of Ophthalmology, University of Basel, Basel 4031, Switzerland; Department of Ophthalmology, Inselspital, Bern University Hospital, Bern 3010, Switzerland; Institute of Molecular and Clinical Ophthalmology Basel (IOB), Basel 4031, Switzerland; Department of Ophthalmology, University of Basel, Basel 4031, Switzerland; Department of Ophthalmology, Instituto de Oftalmologia Dr Gama Pinto (IOGP), Lisbon 1169-019, Portugal; iNOVA4Health, NOVA Medical School, Faculdade de Ciências Médicas, NMS, FCM, Universidade NOVA de Lisboa, Lisbon 1169-056, Portugal; Institute of Molecular and Clinical Ophthalmology Basel (IOB), Basel 4031, Switzerland; Department of Ophthalmology, University of Basel, Basel 4031, Switzerland; Department of Genetics and Genome Biology, University of Leicester, Leicester LE1 7RH, UK; Institute of Molecular and Clinical Ophthalmology Basel (IOB), Basel 4031, Switzerland; Department of Ophthalmology, University of Basel, Basel 4031, Switzerland; Department of Computational Biology, University of Lausanne, Lausanne 1015, Switzerland; Department of Computational Biology, University of Lausanne, Lausanne 1015, Switzerland; Department of Medical Genetics, Hospital Santa Maria, Centro Hospitalar Universitário Lisboa Norte (CHULN), Lisbon 1649-035, Portugal; Department of Medical Genetics, Hospital Santa Maria, Centro Hospitalar Universitário Lisboa Norte (CHULN), Lisbon 1649-035, Portugal; Department of Ophthalmology, Instituto de Oftalmologia Dr Gama Pinto (IOGP), Lisbon 1169-019, Portugal; Department of Medical Genetics, Hospital Santa Maria, Centro Hospitalar Universitário Lisboa Norte (CHULN), Lisbon 1649-035, Portugal; Laboratory of Basic Immunology, Faculty of Medicine, University of Lisbon, Lisbon 1649-028, Portugal; Department of Ophthalmology, Instituto de Oftalmologia Dr Gama Pinto (IOGP), Lisbon 1169-019, Portugal; Institute of Molecular and Clinical Ophthalmology Basel (IOB), Basel 4031, Switzerland; Department of Ophthalmology, University of Basel, Basel 4031, Switzerland; Department of Genetics and Genome Biology, University of Leicester, Leicester LE1 7RH, UK

**Keywords:** inherited retinal diseases, EYS, Portugal, homozygosity

## Abstract

Inherited retinal diseases (IRDs) are a group of ocular conditions characterized by an elevated genetic and clinical heterogeneity. They are transmitted almost invariantly as monogenic traits. However, with more than 280 disease genes identified so far, association of clinical phenotypes with genotypes can be very challenging, and molecular diagnosis is essential for genetic counseling and correct management of the disease. In addition, the prevalence and the assortment of IRD mutations are often population-specific. In this work, we examined 230 families from Portugal, with individuals suffering from a variety of IRD diagnostic classes (270 subjects in total). Overall, we identified 157 unique mutations (34 previously unreported) in 57 distinct genes, with a diagnostic rate of 76%. The IRD mutational landscape was, to some extent, different from those reported in other European populations, including Spanish cohorts. For instance, the *EYS* gene appeared to be the most frequently mutated, with a prevalence of 10% among all IRD cases. This was, in part, due to the presence of a recurrent and seemingly founder mutation involving the deletion of exons 13 and 14 of this gene. Moreover, our analysis highlighted that as many as 51% of our cases had mutations in a homozygous state. To our knowledge, this is the first study assessing a cross-sectional genotype–phenotype landscape of IRDs in Portugal. Our data reveal a rather unique distribution of mutations, possibly shaped by a small number of rare ancestral events that have now become prevalent alleles in patients.

**Significance statement**

Inherited retinal diseases (IRDs) are rare conditions leading to a lifelong visual impairment, caused by mutations in many different genes. Genotypes of patients are extremely variable and may be specific to individual populations. Currently, no comprehensive data exist for the Portuguese. Following the recruitment and assessment of more than 200 families, we describe here the first clinical and genetic landscape of IRDs in Portugal. Our results show a rather unique distribution of genotypes, likely determined by a limited number of ancestral mutations that have become prevalent in contemporary patients. These data contribute to a better description of IRD genetics globally and will serve as a basis for future diagnosis and genetic counseling of patients from this region of Europe.

## Introduction

Inherited retinal diseases (IRDs), including retinitis pigmentosa (RP) and allied diseases, encompass a spectrum of rare disorders characterized by progressive visual impairment, often resulting in legal or complete blindness at the end stage (1). Symptoms and clinical signs vary considerably across patients and constitute the basis for defining specific IRD subclasses. Loss of vision is ultimately caused by the degeneration or dysfunction of photoreceptors (rods and cones), the light-sensing neurons of the retina. This is due, in turn, to mutations in genes that are important for the homeostasis and survival of this class of cells, of the retinal pigment epithelium (RPE), or of other retinal cell types. Patients with RP typically start suffering from night blindness because of the loss of rods, followed by rod- and cone-mediated visual impairment affecting first the mid-periphery and then extending to the periphery and the center of the visual field. In contrast, patients with cone dystrophies generally experience photophobia, decreased central visual acuity, and impaired color vision as a result of the predominant loss of cone photoreceptors. Other commonly diagnosed forms of IRD include cone-rod degenerations, in which first the cone and then the rod systems are affected, as well as Leber congenital amaurosis (LCA), characterized by congenital or infantile blindness. Finally, some IRDs predominantly affect a particular region of the retina, such as the macula in Stargardt disease, or are a component of multiorgan conditions, the most prevalent of which is Usher syndrome, a ciliopathy characterized by the loss of both sight and hearing (2).

IRDs are transmitted as a monogenic trait, i.e. displaying recessive, dominant, X-linked, or mitochondrial inheritance. To date, approximately 280 different genes have already been linked to retinal pathogenesis, making IRDs one of the most heterogeneous Mendelian conditions at the genetic level (3). However, causative mutations remain undetected in approximately one-third of all investigated families (4), indicating that a substantial number of elusive genetic variants or novel disease genes still remain to be discovered. Identifying the molecular culprit for IRDs is clearly crucial to establish an accurate diagnosis and perform proper genetic counseling. Moreover, precise molecular diagnosis is necessary to give patients hope of being enrolled in one of the many gene-based therapy trials that are currently being developed (5, 6).

The genetic landscape of IRDs is in many cases population-specific and it is not uncommon to detect variants that are unique to a particular geographical region (7–15). Currently, there is very limited information on the genetic bases of IRDs in Portugal and no comprehensive data concerning their global clinical prevalence and/or their mutational landscape. In this work, we report the results of a cross-sectional study combining the clinical and molecular assessment of 230 Portuguese families with hereditary retinal diseases.

## Results

### General and clinical features of the cohort

The cohort analyzed was composed of 230 index cases (one per family), including 110 males (48%) and 120 females (52%), as well as 40 affected and 49 unaffected relatives (Table 1). All subjects originated from Portugal. For probands, age at first visit ranged from 3 to 81 years (average: 40 years). Diagnoses were made at the clinical level, and included: retinitis pigmentosa (109 patients, 47%), cone-rod dystrophy (32 patients, 14%), LCA (24 patients, 10%), Usher syndrome (14 patients, 6%), Stargardt disease and other macular dystrophies (13 patients, 6%), cone dystrophy (11 patients, 5%), and other forms of IRDs (27 patients, 12%). The inheritance pattern was ascertained by family history, as reported by the proband. Most cases were isolated (110, 48%), while recessive inheritance of the disease involved 86 probands (37%). Dominant transmission of the disease was present in 17 families (7%), X-linked inheritance was detected in 16 families (7%), and for 1 individual inheritance could not be ascertained.

**Table 1.** General features of the cohort.

**Age of index patient at recruitment (years)**		
<10	9	4%
10–25	42	18%
26–50	104	45%
> 50	75	33%
**Sex**		
Male	110	48%
Female	120	52%
**Inheritance (based on family history)**		
Isolated	110	48%
Autosomal recessive	86	37%
Autosomal dominant	17	7%
X-linked	16	7%
Unknown	1	1%
**Ocular phenotype**		
Retinitis pigmentosa	109	47%
Cone-rod dystrophy	32	14%
Leber congenital amaurosis	24	10%
Usher syndrome	14	6%
Stargardt disease and macular dystrophy	13	6%
Cone dystrophy	11	5%
Chorioretinal dystrophy	9	4%
Syndromic IRD	9	4%
Achromatopsia	4	2%
Congenital stationary night blindness	2	1%
X-linked retinoschisis	2	1%
Other	1	<1%

For 24 patients, the initial clinical diagnosis was revised during the course of the study. These included 15 cases for whom new data emerged from follow-up clinical examinations, four cases for whom the ocular disease was at a very advanced stage and therefore a precise diagnosis could not be made (molecular results were later integrated), four syndromic cases for whom extra-ocular signs were not immediately recognized as part of the same genetic disease, and one case for whom the inheritance pattern was misleading, resulting in an incoherent diagnosis (seemingly dominant Usher syndrome) (Table S1, Redefined Clinical Diagnosis). Of note, LL335 carried biallelic mutations in *TRAF3IP1*, associated so far with Senior-Løken syndrome (16). However, our patient did not report any clear extra-ocular symptoms and no obvious pathological signs were observed, except for microhematuria. Kidney ultrasound imaging revealed potential morphological changes, but renal function was normal. Her family history was negative for other Senior-Løken cases. Although it is likely that future examinations could reveal typical signs of this syndrome, we did not reclassify this case due to a lack of specific clinical information.

Clinical features of all probands are reported in Table S1.

### Molecular findings and global landscape of mutations

**Fig. 1.**
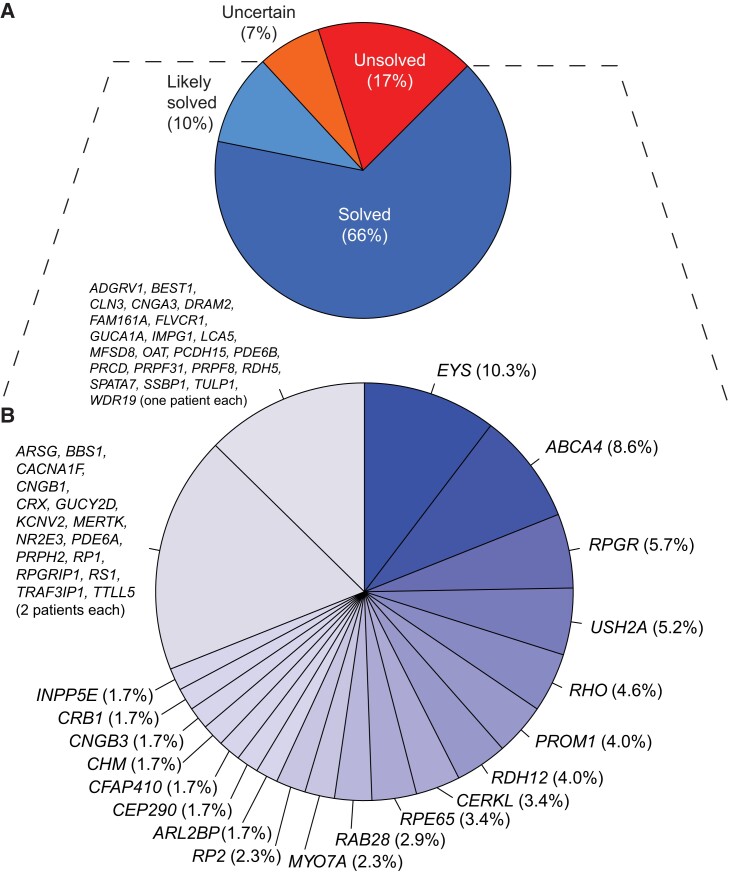
Genetic classification of the cases analyzed. A) Classification of patients by their diagnostic status at the molecular level. Percentages are computed over the total number of patients in the study. B) Further stratification of patients from the “likely solved” and “solved” classes, by disease gene harboring causative mutations, represented by individual slices of the chart. Genes that were mutated in one or two patients were grouped. Percentages are computed over the total number of patients from the “likely solved” and “solved” classes.

Our analysis of single nucleotide variants (SNVs), small insertions/deletions (indels), and copy number variations (CNVs) allowed detecting a total of 157 different variants in 174 index patients within the “likely solved” and “solved” classes (see Methods), of which 34 were never reported before, within a total of 57 genes (Table S2, Fig. 1). The most represented type of variants were SNVs (110 distinct variants, 200 occurrences), followed by small indels (37 distinct variants, 77 occurrences) and CNVs (10 distinct variants, 32 occurrences). SNVs were in turn composed of 66 missense variants, 23 nonsense variants, 19 intronic substitutions affecting splicing, and one start-loss variant. A few synonymous variants were encountered as well, but only one [NM_000329.3:c.1101A>G, p.(Arg367=) in *RPE65*] was considered causative since it interferes with pre-mRNA splicing (17).

The most frequently mutated gene was *EYS*, followed by *ABCA4*, *RPGR*, and *USH2A* (Fig. 1B). This genetic landscape resembled that of other European populations (18, 19) but did not closely match any of them, and was somehow unique because of an elevated prevalence of *EYS*-related retinopathy (see below). Importantly, this landscape was rather different from the one documented in IRD patients from Spain, a neighboring country, where patients with *EYS* mutations represented less than 2% of all cases (8). In addition, the most frequent mutations identified displayed some degree of correlation with the geographical origin of patients and their ancestors, within Portugal (Fig. S1).

Interestingly, we noted that in 5 of the 11 families with index male patients carrying hemizygous mutations in the X-chromosome genes *RPGR* and *RP2*, a considerable number of female relatives (23 in total), obligate or likely carriers of the same mutations, had clinical or subclinical signs and symptoms. In addition, three index patients with mutations in *RPGR* were indeed heterozygous female individuals with RP (Table S2, Fig. S2). These results are in agreement with recent data showing that heterozygosity for X-linked IRD mutations often results in an ocular phenotype in females (20–22).

Overall, the percentage of patients who could be diagnosed at the genetic level was 76% (Fig. 1A), with minor differences across the various diagnostic classes (Fig. S3). General metrics on the molecular genetics of the cohort are provided in Fig. S4.

### Recurrent homozygous mutations

**Fig. 2.**
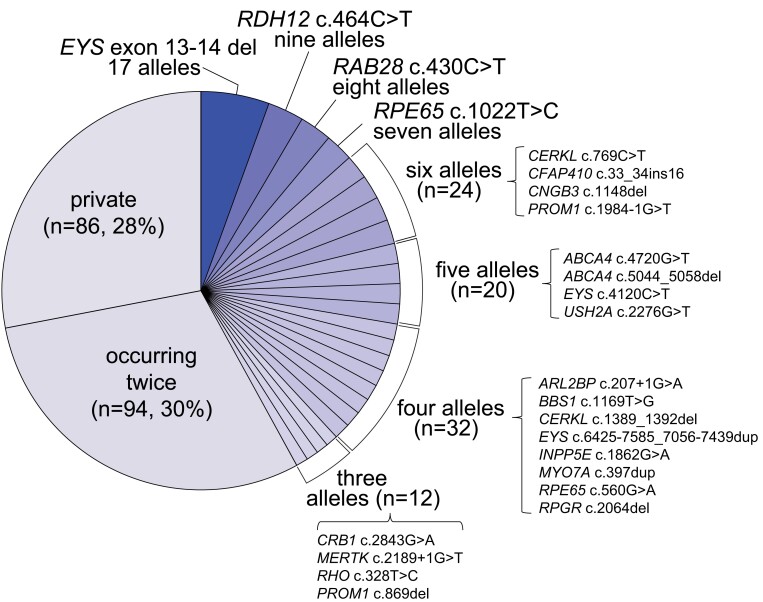
Prevalence of the mutations detected. Individual slices of the chart refer to specific variants, with the exception of mutations occurring only once (private) or twice, which were grouped. *n* refers to the total number of alleles identified in a given group. Percentages are computed over the total number of causative alleles detected in patients from the solved and likely solved classes, regardless of the inheritance mode of the disease. The correct HGVS nomenclature for *“EYS* exon 13-14 del” is *EYS* c.2024-5718_2260-10064del and for “*CFAP410* c.33_34ins16” is *CFAP410* c.33_34insAGCTGCACAGCGTGCA; a simplified notation was used here because of space constraints.

We noticed that, in contrast to other population-based genetic studies, where private mutations typically represent the largest group of variants identified (reaching up to 70% of the total) (8, 10, 11, 15, 18), in our cohort mutations occurring only once accounted for only 28% of all pathogenic or likely pathogenic variants (PLPs, see Methods). Conversely, 30% of mutations were detected twice, while 42% were detected three times or more (Fig. 2).

**Fig. 3.**
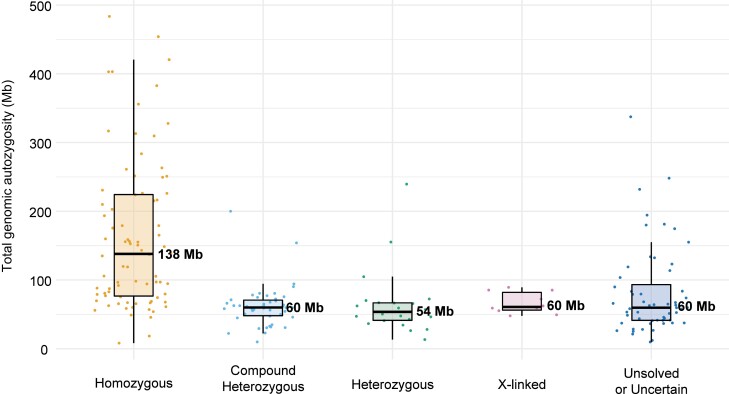
Boxplots of total genomic autozygosity, as a function of the genotypes identified. Levels of autozygosity are expressed as the sum of all homozygous regions detected in each genome, for patients analyzed by NGS procedures. Values relative to individual patients are represented by dots, while median values are indicated by horizontal thick bars, with numbers (Mb: megabases). Standard notation for boxplots applies to other components of the graph. Sixteen index subjects were not analyzed, due to lack of appropriate quality data.

**Fig. 4.**
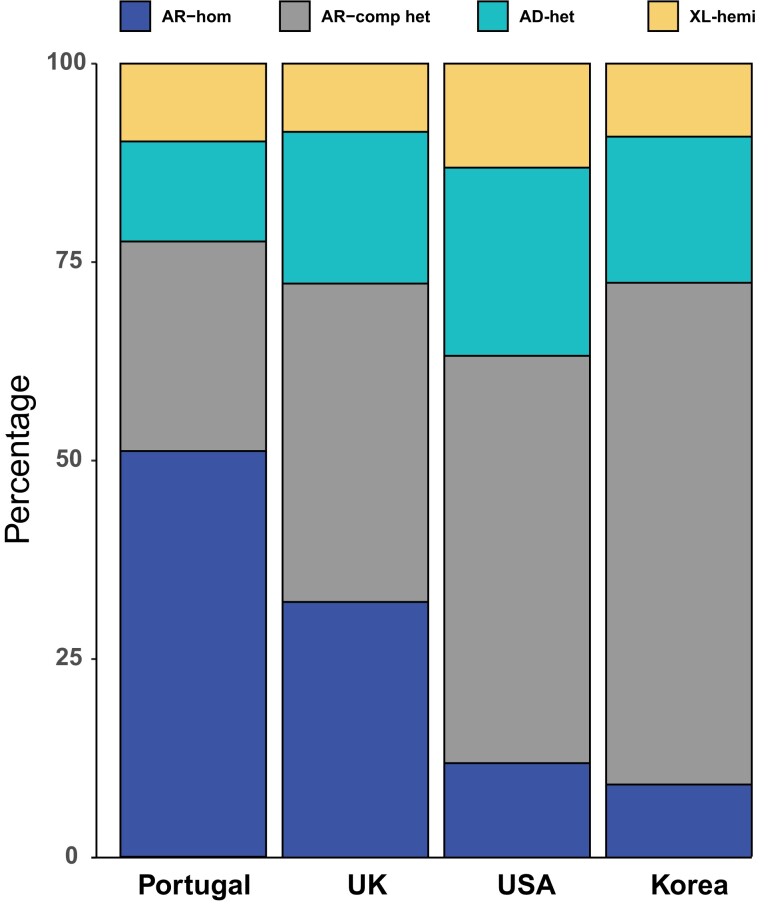
Relative number of patients, by genotype and mode of inheritance of the disease, in our cohort vs. similar studies in other populations. Data from this work (Portugal) are compared with those from three other large studies from the United Kingdom (12), the United States (7), and Korea (10). Our genotypes are enriched in homozygous and reduced in compound heterozygous recessive mutations. AR-hom, autosomal recessive inheritance, mutation in homozygosis; AR-comp het, autosomal recessive inheritance, mutations in compound heterozygosity; AD-het, autosomal dominant inheritance, mutation in heterozygosis; XL-hemi, X-linked inheritance, mutation in hemizygosis (in males).

The analysis of pathogenic genotypes across the whole cohort also revealed a high number of recessive mutations in homozygosis, which correlated, as expected, with overall genome-wide individual autozygosity (Figs. 3 and 4). This particular feature was also highlighted following the comparison of the genotypes detected in our patients with data from similar studies on other populations (Fig. 4). Of note, all recurrent homozygous pathogenic variants occurred within regions of homozygosity (ROHs), with the exception of c.1148del in *CNGB3* in a single patient (Table S3).

To gain insights into these specific findings, we further analyzed the level of autozygosity and inheritance pattern in all solved and likely solved patients, as a function of their date of birth. While there was a progressive decrease in the total size of genomic ROHs in younger vs. older patients, the percentage of cases with homozygous mutations remained relatively constant over time (Fig. S5). Altogether, these data indicate that pathogenicity by homozygosity was likely the consequence of founder mutations and limited endogamy in specific areas, rather than the effect of genetic homogeneity at the population level. Notably, no clear-cut cases of uniparental isodisomy were identified.

The genotypes of four patients with cone-rod dystrophy, all homozygotes for the newly identified mutation p.(His144Tyr) in the gene *RAB28*, fit very well into this category. Our patients belonged to four different pedigrees, although two patients (LL301 and LL252) were found to be distantly related (third cousins once removed; their great-grandfather and great-great-grandfather, respectively, were brothers). Moreover, they all originated from a particular region in the North of the Lisbon district (Fig. S1), a feature indicative of a possible founder effect for their condition. Clinically, they had decreased visual acuity, symptomatic from the first decade of life, temporal optic disk pallor, and abnormal foveal reflex with no pigment (Fig. S6), which is typical for cone and early cone-rod dystrophy. Low visual acuity was noticed between early infancy and eight years of age. All patients were emmetropic at first examination, except for LL317 who was moderately myopic. Our patients at an earlier age had lower visual acuity than the series reported by Iarossi et al. (23), one of the largest and well-characterized cohort of patients with *RAB28* mutations.

Another example is represented by two sisters, LL20 and LL253, both suffering from cone-rod dystrophy and found to carry a novel intronic variant, NM_001349884.2:c.517+5C>A, in *DRAM2*, which was prioritized based on a dbscSNV_ADA score of 0.66 (Table S4). Symptom onset for the index case was 30 years of age, with photophobia. However, low visual acuity was first noticed only at 43 years, and it degraded rapidly. Her sibling presented with a more advanced disease, with strabismus at 18 years of age and low visual acuity since the age of 39 years. At 47 years, she had deeper retinal atrophy and scarce bone spicule peripheral pigmentation. Low visual acuity started later than the median age reported previously in a series of patients with mutations in the same gene (24). None of these patients had intraretinal cysts in the regions of preserved photoreceptors, however, it is possible that these were present in earlier stages of the disease (Fig. S7).

**Fig. 5.**
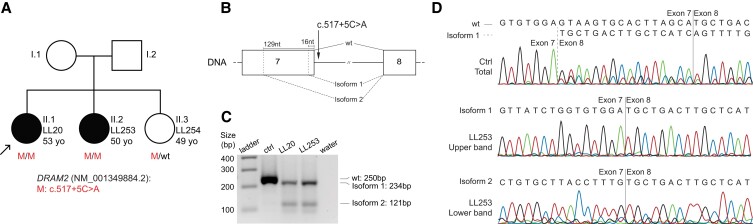
Effects of the *DRAM2* intronic variant c.517+5C>A on pre-mRNA splicing. A) Co-segregation analysis, within LL20's family, of genotypes and phenotypes. B) Schematic representation of exons 7 and 8 of *DRAM2*, including the position of the variant and the main splicing events detected. The canonical mRNA transcript is indicated by “wt”. Isoforms 1 and 2 result from the preferential activation of alternative donor sites, located 16 and 129 nucleotides upstream of the canonical one, respectively. Isoform 1 contains a premature stop triplet at codon 169, whereas isoform 2 bears an in-frame deletion of 43 codons. C) Electrophoresis of RT-PCR products from leukocytes of a control individual (ctrl) and of patients LL20 and LL253. The amplification product of the canonical transcript (wt) is 250 bp in length, while isoform 1 and isoform 2 have sizes of 234 and 121 bp, respectively. water: negative control of amplification. D) Electropherograms of the PCR products shown in (C). The control sample contains a mixture of the canonical mRNA and isoform 1, indicating that the latter transcript is produced, although in minimal quantities, even in the absence of the mutation.

The DNA variant identified homozygously was present in a 4.5Mb region of autozygosity on chromosome 1, detected in LL20, and was predicted to disrupt the donor splice site of intron 7 of *DRAM2*. To assess pathogenicity of this variant, we analyzed RNA from leukocytes of both sisters and found that c.517+5C>A abolished the use of the canonical donor site for intron 7 and favored instead the occurrence of two other splicing events, starting at 16 and 129 nucleotides upstream of this site (Fig. 5). The first isoform was the most represented one and contained a shift of the reading frame, therefore resulting in a nonproductive transcript. The second event was present at a much lower level and resulted in an in-frame deletion of 43 amino acid residues, predicted to be part of the fourth and fifth transmembrane helices of the protein. No wild-type transcript was detected in leukocytes obtained from these patients, in comparison with the control (Fig. 5C,D).

### A deletion in EYS is a common mutational event in our cohort

**Fig. 6.**
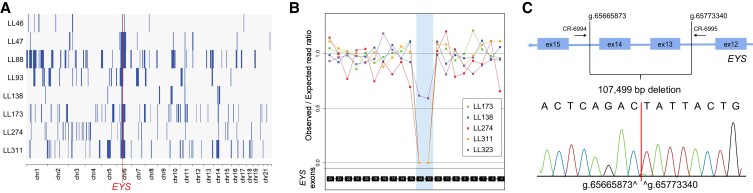
Features of the prevalent deletion of exons 13 and 14 in *EYS*. A) Autozygosity plots of eight patients, showing the common homozygous haplotype on chromosome 6 that eventually led to the identification of the most common mutation from our cohort. Homozygous regions are indicated by solid blue bars, while the red vertical, dotted line shows the minimal critical region spanning *EYS*. B) Coverage plot of four selected homozygotes for this mutation, as well as another patient (LL323, affected sister of index patient LL175), who carries this deletion in compound heterozygosity with another mutation in *EYS*. The area shaded in blue indicates the approximate location of the deletion, as inferred from the reduction of sequence coverage. C) Schematic representation detailing the deletion and the resulting novel junction. Genomic coordinates are given with respect to build GRCh37-hg19 of the human genome sequence. PCR primers allowing the detection of this junction are also indicated.

Homozygosity mapping and unsupervised detection of common haplotypes across all patients highlighted the presence of a ROH of 9.5Mb on chromosome 6 that was shared by eight different families. Molecular analysis showed that this region harbored a deletion of 107.5 kb encompassing exons 13 and 14 of the gene *EYS* (NC_000006.11:g.65665873_65773340del), with breakpoints within introns 12 and 14 (Fig. 6). Overall, this mutation was detected 17 times in nine index patients (eight homozygotes), accounting for as much as 15% (9/60) of all recessive RP cases from our cohort, hence representing a common and possibly a founder mutation in the Portuguese population. The eight patients homozygous for this deletion presented with typical RP with nyctalopia, tubular visual field and generally preserved central visual acuity, except in advanced disease (Fig. S8). Comparison to other patients with RP did not highlight any particular clinical feature specific to this subcohort of cases.

## Discussion

We set out to determine the spectrum of variants causing IRDs in a large cohort of patients from Portugal. Over six years, we analyzed in total 230 index patients and 89 affected and unaffected relatives, all recruited at the Eye Genetics Consultation of the Ophthalmic Institute Dr. Gama Pinto in Lisbon (IOGP). Our analyses led to a genetic diagnosis in 76% of families, which can be considered a high diagnostic success rate according to the most recent literature on genetics of IRDs (e.g. (25, 26)). As for other studies, this high diagnostic yield was the consequence of an integrated approach linking allele frequency from large repositories of controls, *in-silico* predictive tools, a specific assessment of sequence coverage to identify CNVs, as well as accurate clinical characterization. Of note, CNV analysis in this cohort identified as many as ten different pathogenic events, for a total of 32 individual occurrences, representing ∼10% of all mutated alleles. Computer driven and manual inspection of the regions flanking these events revealed no obvious DNA homology that could have promoted recombination events, with the exception of a previously reported *AluSx* repeat (27) at both sides of the deletion in *CLN3*, detected in patient LL244.

Our genetic landscape shows a relatively high number of homozygous mutations. More specifically, ∼51% of index patients with molecular diagnosis carried homozygous recessive mutations. In particular, the top eight most recurrent mutations represented ∼20% of all detected pathogenic alleles. Almost all homozygous mutations also occurred in ROHs, and the few exceptions to this rule may represent false negatives resulting from the low efficiency of WES-based genotyping. In addition, unlike other cross-sectional IRD studies (7, 10, 18), many mutations were present in two or more unrelated cases. Taken together, our data indicate that in our cohort pathogenesis was mostly caused by prevalent recessive founder mutations, benign in heterozygosis and inherited homozygously in patients. This is the case, for instance, of the recurrent deletion of exons 13 and 14 in *EYS*. Previous mutational screens from this and other countries, including Italy, France, and Spain, highlighted the presence of a few deletions in *EYS*, including uncharacterized deletions involving exons 13 and 14 (28–31). It is currently unclear whether the prevalent deletion detected here corresponds specifically to any of these mutations. However, the possibility that this CNV could represent an allele common to various European populations is an intriguing possibility that can now be validated by assessing the presence of the junctional event identified in this study.

Surprisingly enough, at this day the Portuguese population is the only one in Western Europe for which no systematic molecular investigation on IRDs had been performed, apart, to the best of our knowledge, from the genetic screen of 27 individuals with Stargardt disease (32) and of six patients with sector retinitis pigmentosa (31). The need for a better genetic characterization of patients with retinal dystrophies in Portugal has been highlighted previously (33, 34), and has become now particularly relevant since the recent commercialization of an AAV-mediated treatment (35) and the start of several gene-based clinical trials. With our work, we present the first cross-sectional genotype–phenotype study of IRDs in the Portuguese. In addition to identifying many novel pathogenic genotypes, we delineate a population-specific mutational landscape, which can be used to direct patient treatment and design future interventions for this class of conditions.

## Methods

### Families and samples

This study adhered to the tenets of the Declaration of Helsinki and was approved by the Ethics Committees of our respective Institutions (Comissão de Ética para a Saúde do Instituto de Oftalmologia Dr. Gama Pinto, Cantonal Committee of Canton Vaud for Research Activities on Human Subjects, and Ethikkommission Nordwest- und Zentralschweiz). Written informed consent was obtained from all individuals or their legal guardians prior to their inclusion in this study. A total of 319 individuals, including 230 index patients and additional family members, were recruited between 2017 and 2022 at the Ophthalmic Institute Dr. Gama Pinto (IOGP), in Lisbon, Portugal. Affected individuals were assessed based on their medical history and mode of inheritance of the disease in their family, as reported from the proband. All patients underwent a complete and standardized ophthalmological evaluation, including assessment of best corrected visual acuity (BCVA), slit-lamp examination, fundus imaging (center and periphery, with autofluorescence), optical coherence tomography (OCT), electroretinography (ERG), color vision test, and visual field assessment (Goldmann or Humphrey). On this basis, a clinical diagnosis was made at enrollment and, in a small number of cases, a revised diagnosis was established whenever new clinical data would emerge from follow-up examinations (Table S1). The precise clinical features of eight patients, LL1, LL64, LL89, LL105, LL135, LL197, LL235, and LL291 were previously reported in other publications (36–39).

DNA was obtained from whole-blood or saliva samples.

### Whole exome and whole genome sequencing (WES, WGS)

WES was performed at Novogene Co. Ltd. (Cambridge, UK), CeGaT GmbH, (Tübingen, Germany), or at the Institute of Genomics of the University of Tartu (Estonia). There, sequencing libraries were generated using the Agilent SureSelect Human All ExonV6 kit (Agilent Technologies) or the Twist Human Core Exome Plus kit (Twist Bioscience), following manufacturer's protocols. Libraries underwent paired-end sequencing on a Novaseq 6000 (Novogene, CeGaT) or on a HiSeq2500 (Institute of Genomics, Tartu) platform (Illumina), resulting in sequences of 100 or 150 bases. The total output per sample was of at least 12 Gbases, representing an average coverage of >150X in targeted regions and resulting in ∼90% of targeted regions with a coverage higher than 20X. WGS was performed at CeGaT GmbH, by the use of a Novaseq 6000 instrument. Output was of at least 90 Gbases per sample, representing an average coverage of >20X and resulting in ∼75% of the genome with a coverage higher than 20X. Libraries were obtained using TruSeq DNA PCR-Free kit (Illumina).

### Mapping and variant calling

For each sample individually, raw sequence files were mapped to the human genome reference sequence (build hg19/GRCh37), using BWA mem (v0.7.17) (40). Then, the Picard module SortSam (v2.14.0-SNAPSHOT) (41) was used to convert SAM files into BAM files and samtools (v1.10) (42) were applied for indexing them. MarkDuplicates (Picard) (41) identified duplicate reads in these BAMs and base quality score recalibration (BQSR) was obtained with GATK (v4.1.4.1) (43), according to the GATK best practices pipeline (BaseRecalibrator and ApplyBQSR) (44, 45). HaplotypeCaller (GATK, gVCF mode) (43) was then used for variant calling. gVCF files were subsequently merged per sequencing batches with CombineGVCFs (GATK) (43) and one VCF file per batch was produced using GenotypeGVCFs (GATK) (43). Variant recalibration was achieved by using VariantRecalibrator and ApplyVQSR (GATK) (43) in parallel for SNPs and indels. Finally, individual VCF files were created and processed using bcftools (v1.10.2, view and norm functions) (42).

### Variant annotation

ANNOVAR (46) was used to annotate variants with the following metrics: RefSeq notations, allelic frequencies from various databases (gnomAD (47), ESP6500 (48), ABraOM (49), ToMMo (50), and the GME database (51)), as well as outputs from predictors of deleteriousness (dbNSFP, v4.1a (52) and MutScore (53)) (Table S4). Gene annotations, such as links to known human (54) and murine (55) phenotypes, DOMINO (56) scores, gnomAD metrics, in-house expression data, as well as quality metrics from the VCF file, were added by the use of simple scripts, developed for this purpose. Finally, the output of splicing predictions such as MaxEntScan (57), dbscSNV ADA and RF (58), and SpliceAI (59) were added to the annotation. At the end of the process, every variant was annotated with more than 200 different metrics.

The resulting variants were then prioritized based on their quality, allelic frequency in population databases, molecular profile (nonsense, frameshift, missense, and splice sites) and, finally, according to compatible patterns of inheritance [i.e. a homozygous or compound heterozygous state for recessive, a heterozygous state for dominant, or a hemizygous (in males) or heterozygous state (in females) on the X chromosome, for X-linked inheritance]. The presence of such variants was first assessed within the list of genes associated with IRDs in the RetNet database (July 2022 release) (3), then extended to OMIM (July 6, 2022 release) entries (54), and finally to the rest of the genes of the human genome.

### Detection of mobile element insertions and CNVs

Scramble (60) and MELT (61) were used to detect mobile element insertions and small intraexonic deletions or insertions. The ExomeDepth software was used to detect CNVs from WES coverage data, with a minimal size of one exon and no maximal size (62). The Mosdepth tool was used to calculate genome-wide sequencing coverage (63). Regions flanking detected CNVs were screened for homologies using RepeatMasker (64). For data from SNP arrays, Log R Ratio (LRR, normalized total intensity) and B Allele Frequency (BAF, allelic intensity ratios) from PLINK were used for large CNV detection. Visualization was achieved by using PennCNV (65) and R (66, 67).

### Variant and patient classification

Previously described variants were classified as pathogenic (P), likely pathogenic (LP), of unknown significance (VUS), benign (B) or likely benign (LB), based on their classification in the ClinVar database (68). Novel variants were manually inspected and assessed for causality based on existing literature, their frequency in the general population (MAF < 1%), established phenotype-genotype association (according to OMIM, RetNet, and/or existing literature presenting adequate evidence), matching inheritance pattern, and suggestive bioinformatic prediction or functional evidence, following the recommendations of the American College of Medical Genetics (ACMG) (69). Results obtained from the *in-silico* prediction tools for all novel missense, intronic and non-frameshift variants are shown in Table S4. All identified variants were then classified according to standard ACMG nomenclature using the Varsome (https://varsome.com) or the Franklin (https://franklin.genoox.com) websites (Table S2, Column M), including manual review of cases for whom segregation was performed. Concerning VUS, only variants that had characteristics similar to those of typical mutations, i.e., (i) had a frequency of less than 0.01 in population databases and an internal inventory, (ii) had an impact at the protein level, (iii) were very conserved (Genomic Evolutionary Rate Profiling, GERP > 4) (70) or had predicted effects on splicing, were considered. All variants were validated using VariantValidator (71) and written in accordance with Human Genome Variation Society (HGVS) nomenclature (72).

At the end of all genetic analyses, each patient was classified as “solved”, “likely solved”, “uncertain”, or “unsolved”. Solved cases included individuals carrying one of the following assortments of P or LP variants (from now on, pathogenic or likely pathogenic variants will be abbreviated as PLP): one heterozygous variant and a condition with dominant inheritance; two variants demonstrated to be *in trans* and a recessive or isolate condition; one variant on the X chromosome, in a male patient with an X-linked disorder. Likely solved patients included individuals with recessive conditions and either one of the following genotypes: a PLP and a VUS demonstrated to be *in trans* or two PLP variants that could not be ascertained to be *in cis* or *in trans* with respect to each other. Patients with a recessive disease were categorized as uncertain when any of the following conditions applied: two VUS demonstrated to be *in trans*; one PLP and one VUS that could not be ascertained to be *in cis* or *in trans*; two VUS that could not be ascertained to be *in cis* or *in trans*; one VUS in a homozygous state. Patients with a dominant or an X-linked condition were categorized as uncertain whenever they had one heterozygous VUS in an autosome or a male patient had a hemizygous VUS on the X chromosome, respectively. Patients with genotypes that did not satisfy any of these conditions (e.g. individuals with a single PLP in a recessive gene) were classified as unsolved.

### SNP array genotyping

DNA samples were genotyped at the iGE3 Platform, University of Geneva, Switzerland, using Illumina arrays (Infinium GSA-24v1.0, GSAMD-24v2.0, GSAv2, CoreExome-24v1.1, and CoreExome-24v1.2), according to the manufacturers’ protocols. Genotype values were called using GenomeStudio (Illumina) and exported in PLINK format (73).

### Targeted sanger sequencing

Primer3Plus (74) was used to design primers for polymerase chain reactions (PCR), performed using the GoTaq polymerase (Promega) and 2 ng of template DNA, according to the manufacturers’ protocol. For *RPGR*-ORF15 sequencing, Phusion High-Fidelity DNA polymerase (ThermoFisher) was used with 100 ng of DNA, and the following primer pair: 5′-GACTAAACCCATAATATCCAAATCCA-3′ (CR-05472); 5′-GCCAAAATTTACCAGTGCCTCCTAT-3′ (CR-05473), enabling the amplification of a 1953bp fragment. All PCR products were treated with ExoSAP-IT (ThermoFisher) and Sanger sequencing was performed by Fasteris SA (Geneva, Switzerland) or by Microsynth (Balgach, Switzerland). Sequences were visualized and compared to the gene's reference sequence (Ensembl (75), GRCh37) with the CLC Genomics Workbench 12 software (QIAGEN).

### Homozygosity mapping

ROH were detected from SNP-array genotype data by using PLINK (73), and from WES data by using AutoMap (76).

### RNA analysis

Assessment of the effect of the NM_001349884.2:c.517+5C>A mutation in *DRAM2* was performed as follows. Three milliliters of peripheral blood were collected from patients and a healthy control in Tempus Blood RNA tubes and total leukocyte RNA was extracted with the Tempus Spin RNA Isolation kit (Applied Biosystems), according to manufacturer's instructions. Two micrograms of RNA were used as a template for cDNA synthesis, by the use of random primers and the MultiScribe Reverse Transcriptase from the High Capacity cDNA Reverse Transcription Kit (Applied Biosystems). RT-PCRs were obtained by using primers 5′-AACAACCCTTTTTGCTGCAC-3′ (CR-7950) and 5′-GGGGTTCCAATGGAGTTTCT-3′ (CR-7951) lying on *DRAM2* exons 7 and 8, respectively, according to standard cycling conditions. Resulting PCR products were resolved on agarose gels and sequenced by the Sanger technique, following their extraction and purification by the GenElute Gel Extraction Kit (Sigma-Aldrich).

### Identification of the EYS deletion breakpoints (exons 13 and 14)

To ascertain the presence of this mutation and identify the precise position of the resulting genomic junction, we performed a PCR with primers on both sides of the breakpoint. In control individuals, the distance between these primers (∼108 kb) is too long to yield a PCR product, whereas amplification of DNA from heterozygous or homozygous carriers of the deletion would result in a 252 bp product. For this reaction, we used primers 5′-CCCCAGCACTCAGAACCATT-3′ (CR-6994) and 5′-GGATCAGACACCTTTTGGCC-3′ (CR-6995), with standard cycling conditions (an initial step of 94 C for 3′, followed by 25 cycles of 94 C for 30″ 58 C for 30″, and 72 C for 30″, and finally a single 5′ elongation step at 72 C).

## Supplementary Material

pgad043_Supplementary_Data

## Data Availability

All data are included in the manuscript and/or supporting information.
