# Supplementary material for: The first genetic landscape of inherited retinal dystrophies in Portuguese patients identifies recurrent homozygous mutations as a frequent cause of pathogenesis
Source: PNAS Nexus. 2023 Feb 13;2(3):pgad043. doi: 10.1093/pnasnexus/pgad043 (PMC10003751; doi:10.1093/pnasnexus/pgad043)
Supplement: pgad043_Supplementary_Data [file pgad043_supplementary_data.zip › PNASNEXUS-PNASNEXUS-2022-00861-s05.pdf]

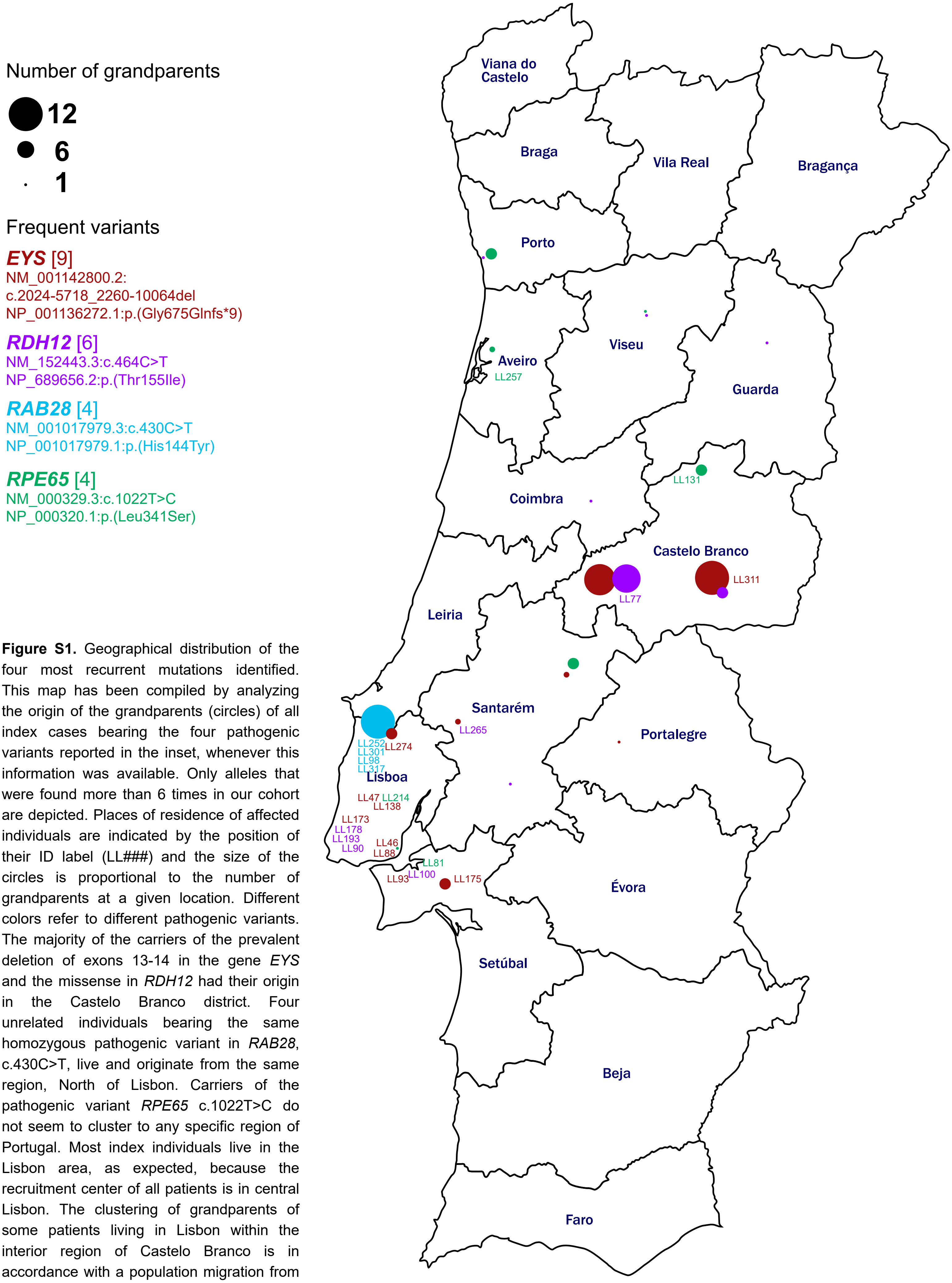

Figure S1. Geographical distribution of the four most recurrent mutations identified. This map has been compiled by analyzing the origin of the grandparents (circles) of all index cases bearing the four pathogenic variants reported in the inset, whenever this information was available. Only alleles that were found more than 6 times in our cohort are depicted. Places of residence of affected individuals are indicated by the position of their ID label (LL###) and the size of the circles is proportional to the number of grandparents at a given location. Different colors refer to different pathogenic variants. The majority of the carriers of the prevalent deletion of exons 13-14 in the gene *EYS* and the missense in *RDH12* had their origin in the Castelo Branco district. Four unrelated individuals bearing the same homozygous pathogenic variant in *RAB28*, c.430C>T, live and originate from the same region, North of Lisbon. Carriers of the pathogenic variant *RPE65* c.1022T>C do not seem to cluster to any specific region of Portugal. Most index individuals live in the Lisbon area, as expected, because the recruitment center of all patients is in central Lisbon. The clustering of grandparents of some patients living in Lisbon within the interior region of Castelo Branco is in accordance with a population migration from interior rural areas to the Lisbon metropolitan area in the 1960-70s.

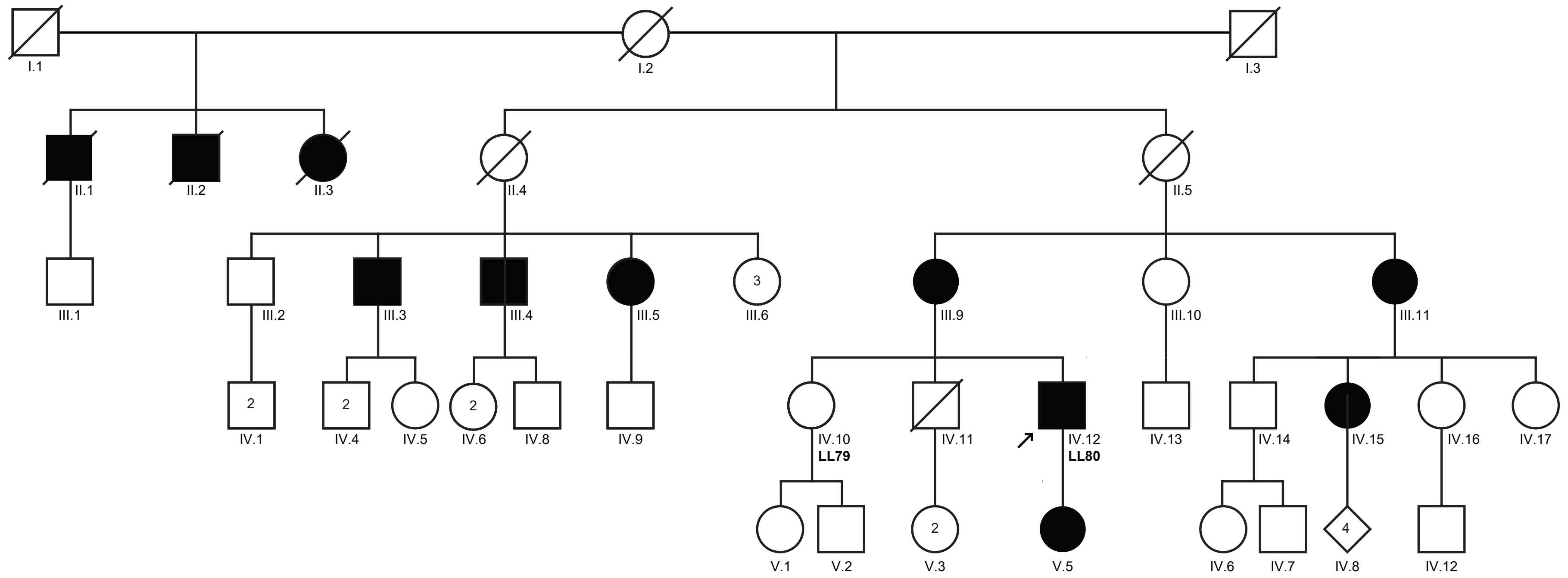

Figure S2. Pedigree of index case LL80, with a hemizygous mutation in *RPGR*. This tree is provided as an example of the five families with male patients with mutations in *RPGR* or *RP2* that also displayed seemingly dominant inheritance, by pedigree analysis alone. All pathogenic phenotypes (RP, in all cases), except for LL80 and his daughter, were ascertained by family history.

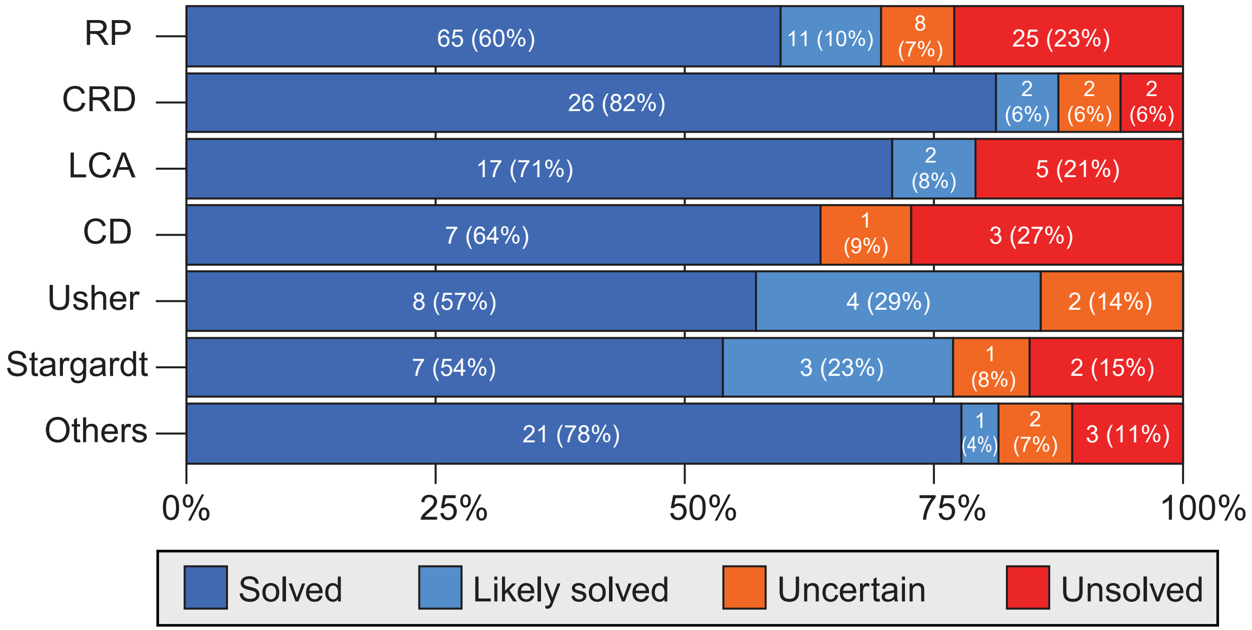

Figure S3. Molecular diagnostic rate in various IRDs classes. Phenotypes that were present in less than 5% of patients are collectively shown as "Others". RP- retinitis pigmentosa, CRD- cone-rod dystrophy, LCA- Leber Congenital Amaurosis, CD- cone dystrophy, Usher- Usher syndrome (all types), Stargardt- Stargardt disease and other macular dystrophies.

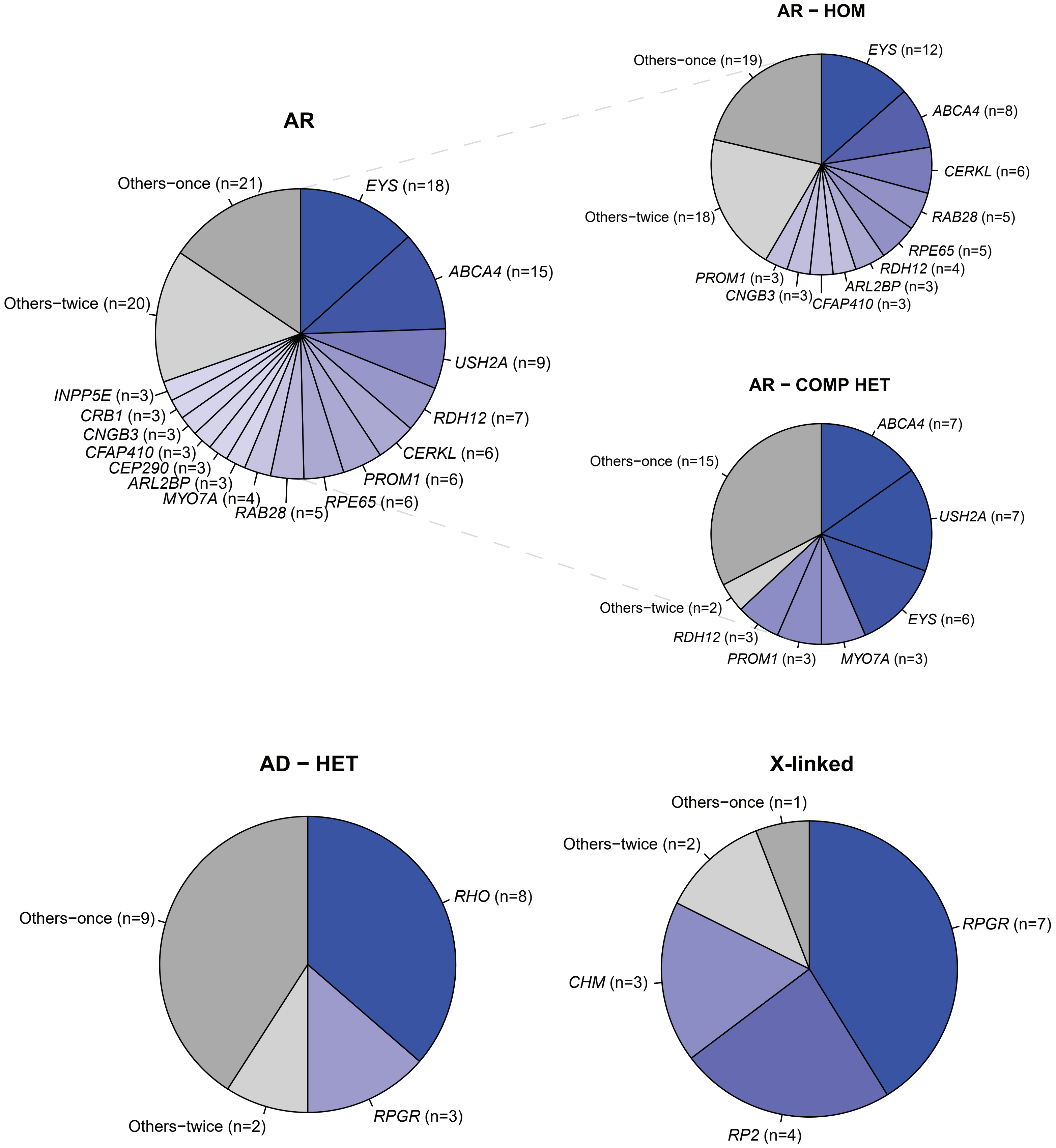

Figure S4. Stratification of patients by mutated gene and inheritance pattern. Slices of pie charts are proportional to the number of individuals found to be positive for mutations in a given gene, which is indicated. Genes associated with disease in less than three people are grouped together and displayed by shades of grey. n refers to the number of patients. AR- autosomal recessive inheritance, all genotypes; AR-HOM, autosomal recessive inheritance, mutation in homozygosis; AR-COMP HET, autosomal recessive inheritance, mutations in compound heterozygosis; AD-HET, autosomal dominant inheritance, mutation in heterozygosis; XL-linked, on chromosome X, mutation in hemizygosis in males.

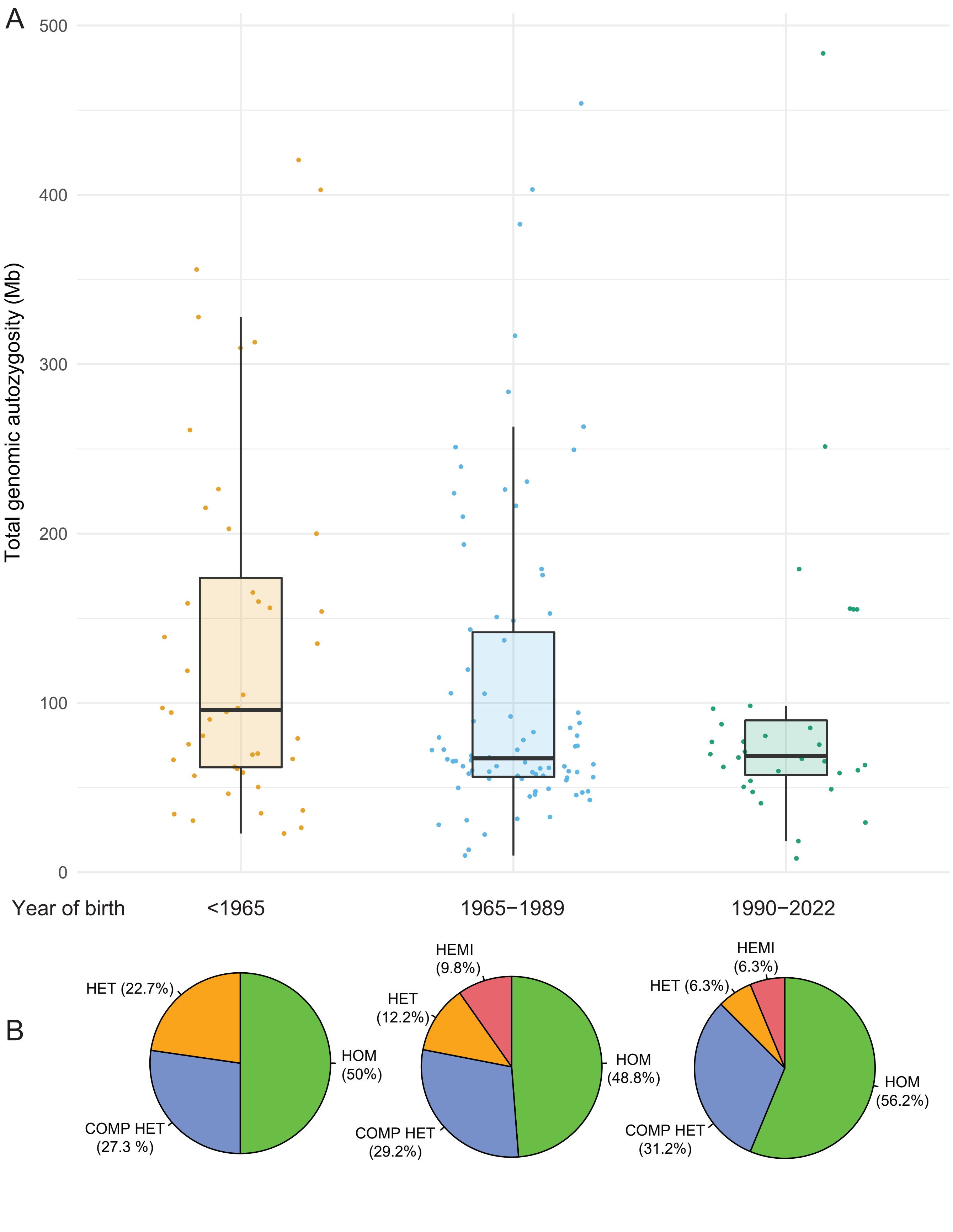

Figure S5. Genomic homozygosity and pathogenic genotypes, as a function of patients' age. A. Boxplots showing the sum of all autosomal ROHs in solved and likely solved patients, stratified by their year of birth. Dots represent individual patients, while median values for each of the three age groups are indicated by the horizontal thick bars. Standard notation for boxplots applies for other components of the graph. B. Genotypes for the same patients. HOM, recessive mutations in homozygosis; COMP HET, recessive mutations in compound heterozygosis; HET, dominant mutation in heterozygosis; HEMI, X-chromosome mutations in hemizygosis (in males).

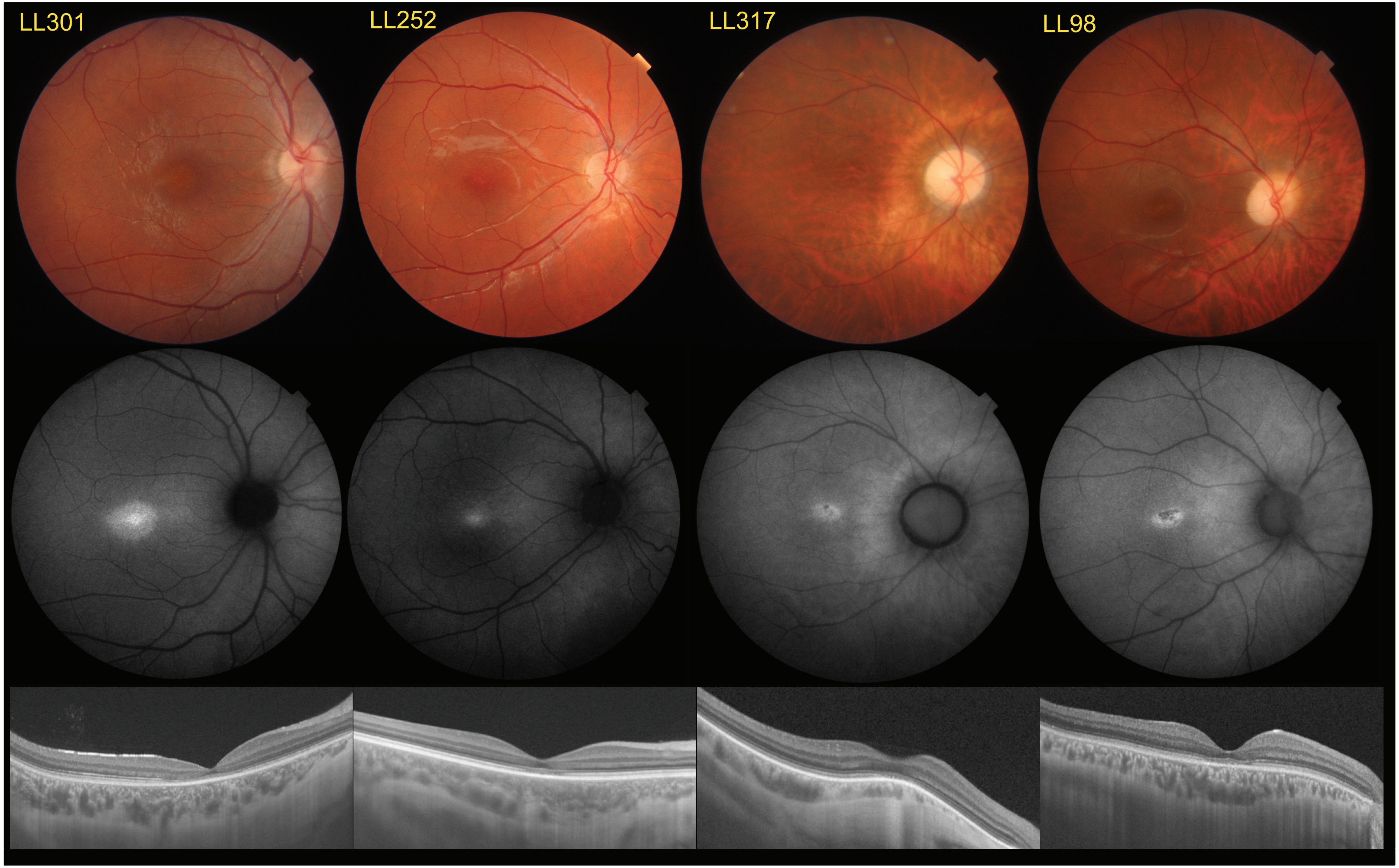

Figure S6. Fundoscopy and OCT of four patients with homozygous c.430C>T mutations in *RAB28*. Top row: posterior pole retinography of the right eye of 4 patients, showing abnormal foveal reflex in all cases. Middle row: fundus autofluorescence revealing foveal hyperautofluorescence. Bottom row: macular horizontal OCT scans demonstrating loss of foveal external layers. Pictures were arranged by patients' ages at examination (LL301, 12 years VA OD 0.6; LL252, 14 years, VA OD 0.2; LL317, 29 years, VA OD 0.13; LL98, 31 years, VA OD 0.3).

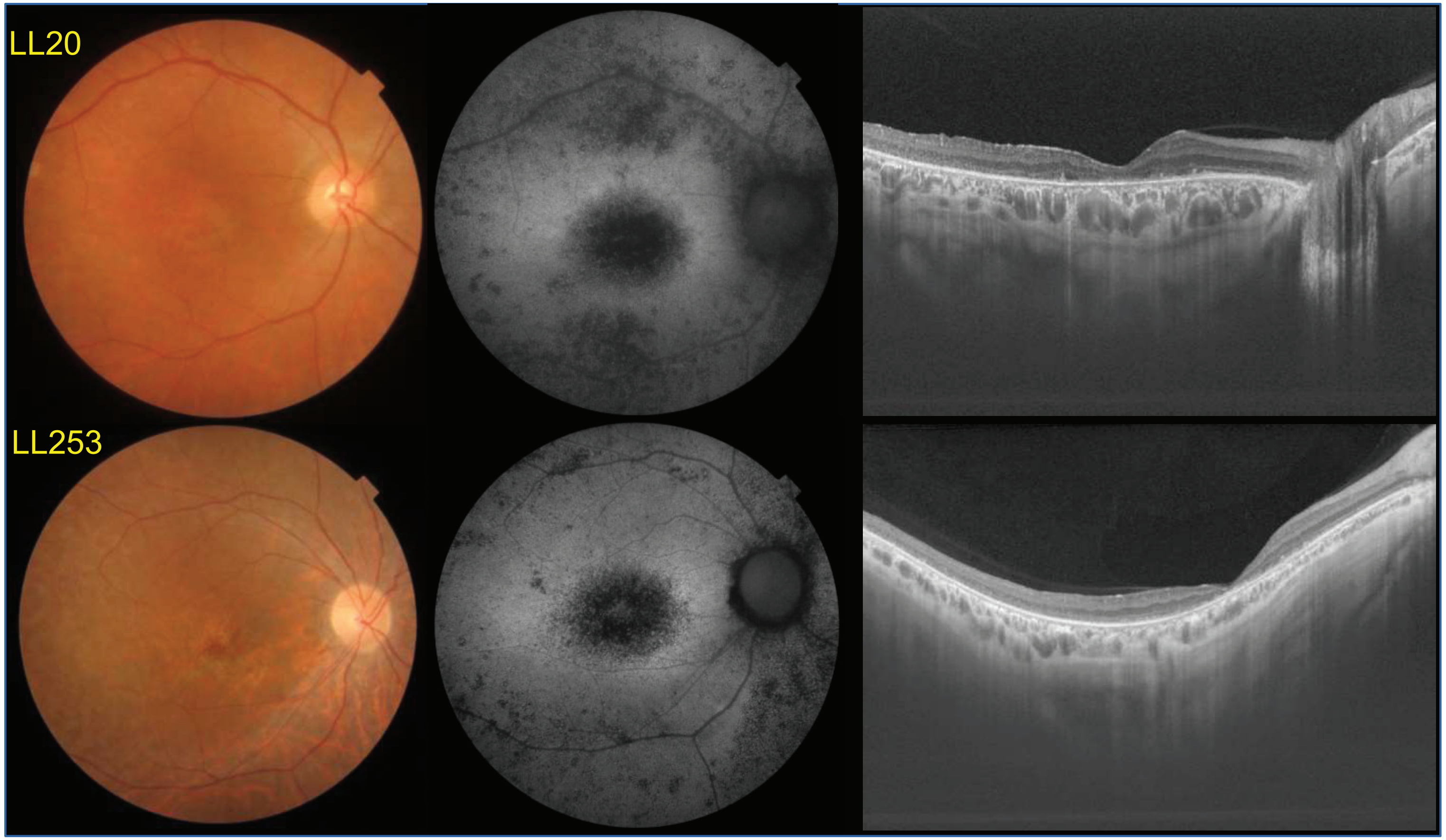

Figure S7. Fundoscopy and OCT of two siblings with homozygous c.517+5C>A mutations in *DRAM2*. Posterior pole retinography (left column) of the right eye of the index patient LL20 (taken at age 47 years) and her affected sister LL253 (also taken at age 47 years), with corresponding autofluorescence (middle column) and OCTs (right column). LL20 presented with 0.1 VA OU and fundoscopy revealed patchy atrophy in the macular area and outside the temporal arcades, expanding into mid periphery, with no pigmentation. Her sister presented with VA 0.08 OD and 0.05 OS, moderately narrowed retinal vessels, deeper retinal atrophy with the same pattern, and scarce bone spicule peripheral pigmentation. In both cases, retinography showed perifoveal atrophy with an external ring of relative sparing and atrophy over the temporal arcades. FAF complements this observation by showing hypoautofluorescent atrophic areas.

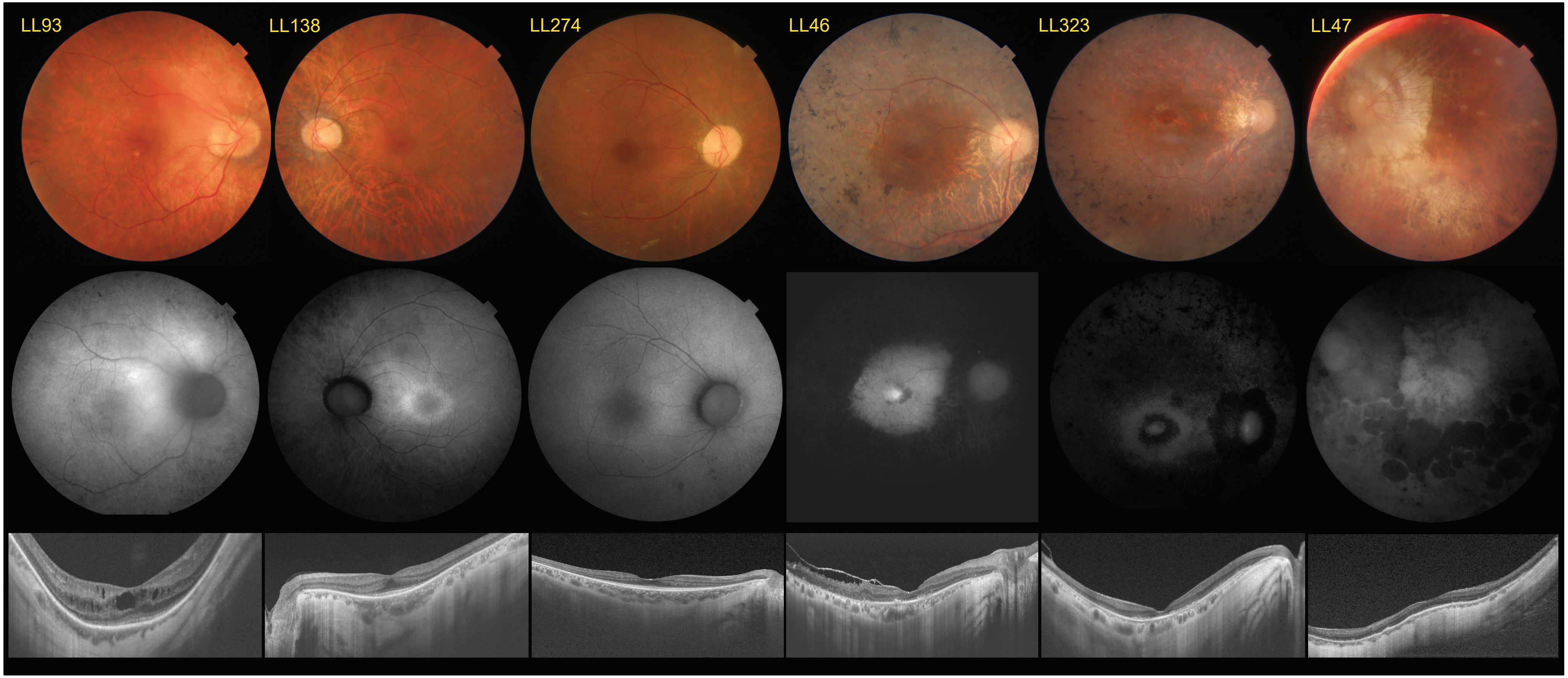

Figure S8. Fundoscopy and OCT of six patients with homozygous deletions of exons 13 and 14 in *EYS*. Posterior pole retinography of six out of eight patients with homozygous deletions in *EYS*, identified by their study IDs (top row) and corresponding autofluorescence (middle row). Macular horizontal OCTs are displayed in the bottom row. Pictures were arranged according to the age of patients at examination (LL93, 34 years; LL138, 39 years; LL274, 45 years; LL46, 48 years; LL323, 60 years; LL47, 64 years). LL93 (BCVA OD 0.5) has high myopia with peripheral visual field changes noticed since 18 years of age. Her retinography showed tessellated fundus and OCT revealed macular oedema, loss of outer layers with relative sparing of central fovea. LL138 (BCVA OS 0.8), also having high myopia and tessellated fundus, with FAF showing ring of hyperautofluorescence around the fovea. He has preserved central foveal layers on OCT. LL274 (BCVA OD 0.8) has a larger area of central sparing, visible on OCT. LL46 (BCVA OD 0.3) presented a well demarcated area of relative retinal preservation at the posterior pole, surrounded by atrophy and moderate pigment clumping. Inferiorly and nasally to the fovea a deeper incomplete circle of atrophy can be seen. Retinography findings are highlighted on FAF, where hypoautofluorescence identifies atrophic areas. LL323, sister of index patient LL175, presented marked vessel narrowing, and a pattern similar to LL46 with more advanced disease. LL47 (BCVA OS 0.6) is a highly myopic patient with RP and diffuse chorioretinal atrophy temporally and inferotemporally to the optic disk.
